# Mapping Protein–Protein Interactions of the Resistance-Related Bacterial Zeta Toxin–Epsilon Antitoxin Complex (ε_2_ζ_2_) with High Affinity Peptide Ligands Using Fluorescence Polarization

**DOI:** 10.3390/toxins8070222

**Published:** 2016-07-16

**Authors:** María Isabel Fernández-Bachiller, Iwona Brzozowska, Norbert Odolczyk, Urszula Zielenkiewicz, Piotr Zielenkiewicz, Jörg Rademann

**Affiliations:** 1 Institute of Pharmacy, Pharmaceutical and Medicinal Chemistry, Freie Universität Berlin, Königin-Luise-Str. 2+4, 14195 Berlin, Germany; isabelfernandezb77@yahoo.es; 2 Institute of Biochemistry and Biophysics, PAS, Pawińskiego 5a, 02-106 Warszawa, Poland; iwonab@ibb.waw.pl (I.B.); nodolczyk@ibb.waw.pl (N.O.); ulazet@ibb.waw.pl (U.Z.); 3 Faculty of Biology, Warsaw University, Miecznikowa 1, 02-106 Warszawa, Poland

**Keywords:** protein–protein interactions, toxin–antitoxin system, drug discovery, bacterial resistance, fluorescence polarization

## Abstract

Toxin–antitoxin systems constitute a native survival strategy of pathogenic bacteria and thus are potential targets of antibiotic drugs. Here, we target the Zeta–Epsilon toxin–antitoxin system, which is responsible for the stable maintenance of certain multiresistance plasmids in Gram-positive bacteria. Peptide ligands were designed on the basis of the ε_2_ζ_2_ complex. Three α helices of Zeta forming the protein–protein interaction (PPI) site were selected and peptides were designed conserving the residues interacting with Epsilon antitoxin while substituting residues binding intramolecularly to other parts of Zeta. Designed peptides were synthesized with an N-terminal fluoresceinyl-carboxy-residue for binding assays and provided active ligands, which were used to define the hot spots of the ε_2_ζ_2_ complex. Further shortening and modification of the binding peptides provided ligands with affinities <100 nM, allowing us to determine the most relevant PPIs and implement a robust competition binding assay.

## 1. Introduction

Antibiotic-resistant strains of pathogenic bacteria have emerged as a worldwide health problem over the past few decades [1,2,3,4,5,6]. As a result, infectious diseases affecting the human population, which are difficult to treat with currently available medications, have become a serious challenge for medicine [7,8]. The situation is worsened by the fact that the development of novel antimicrobial agents has dramatically declined over the past 30 years [9]. Thus, the ability of pathogenic species to respond quickly and effectively to the current treatments creates an urgent medical need to discover new methods of pharmacological intervention based on newly identified cellular targets [8,10,11,12].

Bacterial toxin–antitoxin (TA) systems constitute one class of potential new drug targets against pathogenic bacteria. These systems are frequently found in bacteria and are notably abundant in pathogenic strains [13,14,15]. The TA systems are genetic modules comprising two components: a stable toxic molecule and unstable cognate inhibitory element that prevents the lethal action of the toxin. In normally growing cells, no evident effects of the TA systems are observed. However, different stress conditions lead to the activation of TA systems: non-neutralized toxin inhibits cell growth by targeting essential cellular processes [16,17]. Therefore, several methods of artificially activating the toxin, including the targeting of toxin–antitoxin interactions, have been considered for the potential exploitation of TA systems as an antibacterial strategy [18,19,20,21]. Attempts to find a molecule specifically abolishing TA interactions have been undertaken in the last decade. In 2010, Agarwal and co-workers showed that short peptides can disrupt interactions between toxin–antitoxin proteins [22]. They tested the efficacy of several peptides designed to mimic the two most likely interaction sites of the PemI–PemK proteins of the TA system from *Bacillus anthracis* and 10%–35% inhibition of the PemI–PemK interaction was accomplished with 2 µM of an octapeptide ligand. In the study of Agarwal et al., disruption of the PemI-PemK TA system was obtained by peptide ligands targeting the toxin protein (PemK). In 2015 Lee and co-workers designed peptides that mimic the helical regions of the VapB30 or VapC30 (TA system from *Mycobacterium tuberculosis*) in the heterodimer interface of the VapBC30 complex, capable of disrupting the interactions within the VapBC30 complex in vitro [23]. Also in 2010 Lioy and co-workers published screening results of several libraries of mixed artificial peptides as potential inhibitors of the interactions between Epsilon and Zeta proteins. The study, however, did not provide conclusive results. When the number of peptides of the sub-libraries rendering the positive hits was reduced, the decrease in the BRET signal (proof of disruption of the Epsilon–Zeta interaction) was lost [24].

In this study, we will focus on the toxin–antitoxin system comprising the toxin Zeta (ζ) and the antitoxin Epsilon (ε). The εζ-operon, originally discovered on the pSM19035 plasmid from the clinical *Streptococcus pyogenes* strain [25], is one of the best described TA systems in Gram-positive bacteria [26,27]. It is frequently found on plasmids [28,29,30,31] or on the chromosomes of human pathogens [32,33,34]. The presence of this TA system in the plasmid ensures its stable maintenance in bacterial populations. However, as genes that are responsible for macrolide resistance are present on the same plasmid, the εζ system ensures bacterial resistance to commonly used antibiotics as well.

The Zeta toxin is a protein with kinase activity that directly affects cell wall synthesis by phosphorylating the peptidoglycan precursor (uridinediphosphate-*N*-acetylglucosamine, UNAG), consequently leading to cell lysis [35]. The Epsilon and Zeta proteins form the heterotetramer ε_2_ζ_2_, in which the toxic activity of Zeta is neutralized by direct contact with the Epsilon antitoxin. The molecular basis for the function of the εζ system is the difference in the half-life times of these two proteins [36]. While the Zeta toxin is stable, the free Epsilon antitoxin is a labile protein that is vulnerable to degradation by cellular proteases [37]. Under normal cellular conditions, excess antitoxin stabilizes the TA complex, thereby neutralizing the Zeta toxin. However, when the cellular level of the antitoxin decreases (e.g., upon loss of the plasmid), the toxin protein can no longer be neutralized and then acts on its specific cellular target, leading to bacterial cell death. Considering this functional scenario, we reasoned that disruption of protein–protein interactions in the ε_2_ζ_2_ complex could cause the release of Zeta toxin, thereby killing bacterial cells.

While protein–protein interactions (PPIs) play a key role in the organization of a plethora of biological processes, including conditions of pathological disease states, the disruption of protein–protein binding by small molecules is a considerable challenge for medicinal chemistry [38,39,40]. Nevertheless, rapid progress in this field has been observed in recent years, and several drugs targeting PPIs have been successfully introduced into the market [41,42,43], and more drug-like candidates that target PPIs are currently in clinical trials [44]. A detailed structural knowledge of complexes of interacting protein partners is very helpful for better understanding the functions of PPIs and for the development of PPI inhibitors (PPIIs) [45,46,47]. Based on structural data of protein–protein interactions, peptidic and non-peptidic inhibitors of proline-recognition domains, PDZ domains, SH2 domains, and helix–helix interactions have been developed [48,49,50].

Peptides derived from native protein–protein interaction sites are valuable tools to probe protein–protein interfaces for the identification of “hot-spot” areas and have proven to be excellent starting points for the design of efficient PPI modulators [50,51,52]. The hot-spot regions are formed by residues localized on protein–protein interfaces that have a major impact on complex stabilization and confer most of the free energy of bindings [51,52,53]. Therefore, hot-spot regions are preferred target sites for protein–protein interaction inhibitors.

As the ε_2_ζ_2_ complex structure has been solved by crystallography, a rational starting point for the development of molecules disrupting formation of the complex was available [54].

In this article, we describe the design and development of peptides that are potent ligands of the Epsilon protein. Three α-helices of the Zeta protein that form the binding interface with Epsilon have been selected and were used as the starting point for the development of Epsilon-Zeta interaction disruptors. Potential Zeta-derived peptide ligands were labeled with a fluorophore, and binding of the peptides to Epsilon was determined by a fluorescence polarization assay [55]. Peptide mapping of the interaction surfaces of the ε_2_ζ_2_ complex was expected to define the hot-spots of the binding interface, i.e., amino acids that are essential for binding and that significantly contribute to the binding energy of the complex. Finally, short peptide ligands were identified, and a competitive binding assay was established, allowing for the detection of non-fluorescent binders of the hot-spot area of the complex in high throughput formats. This assay will enable the discovery of non-peptidic, drug-like molecules that are able to bind to the Epsilon protein and that could act as inhibitors of ε_2_ζ_2_ complex formation. Thus, we expect to find new candidates for resistance-free antibiotics, with a novel mechanism of action.

## 2. Results and Discussion

### 2.1. The Design of Peptides as Potential Disruptors of the Epsilon(ε)–Zeta(ζ) Interaction

**Figure 1:**
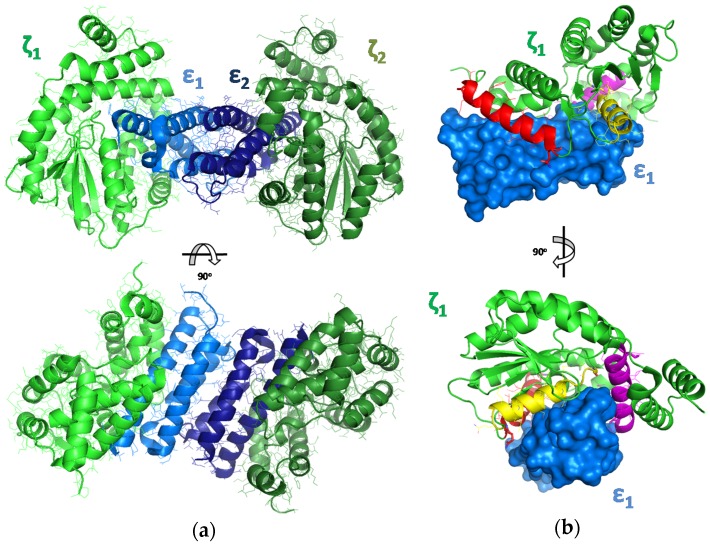
(**a**) The crystal structure of the Epsilon–Zeta tetramer complex ε_2_ζ_2_ (PDBid: 1GVN). The structure is colored by chain: blue/light blue for ε1 and ε2 and green/light green for ζ1 and ζ2. (**b**) The structure of the ε_2_ζ_2_ hetero-dimer complex, with a schematic representation of the fragments selected for further evaluation (peptide I—red, peptide II—yellow, peptide III—magenta).

To propose peptides that could effectively disrupt interactions between the Zeta and Epsilon subunits, we used a template-based modeling approach. As a starting point, a crystal structure of the ε_2_ζ_2_ TA complex (PDBid: 1GVN) derived from the plasmid pSM19035 of *S. pyogenes* [54] was analyzed. The biologically inactive complex forms a stable tetramer (ε_2_ζ_2_)—where a central part consists of a homodimer of two Epsilon antitoxin subunits that is sandwiched by two Zeta toxin subunits (Figure 1) [26,54].

**Figure 2:**
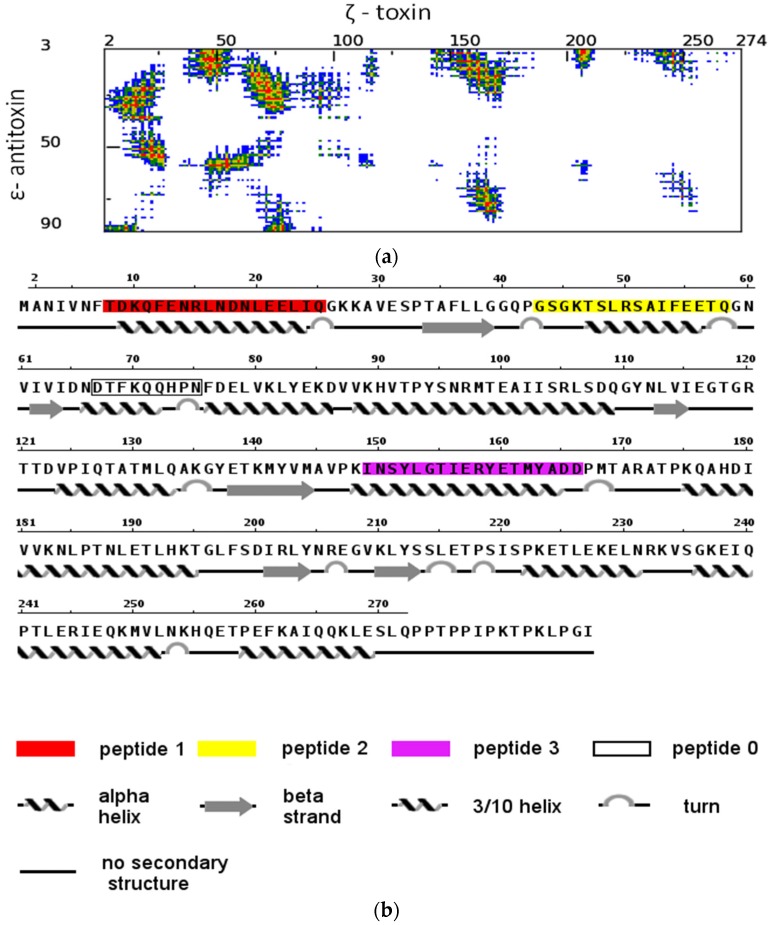
Template-based modelling of potential disruptors of the Epsilon-Zeta interaction. (**a**) Distance range map of the inter-molecular contacts between Epsilon–Zeta interface residues: red, yellow, green, and blue indicate contacts within 7 Å, 10 Å, 13 Å and 16 Å, respectively (generated by the COCOMAPS server). (**b**) The amino acid sequence of the Zeta protein, with a description of secondary structure elements and indicators of the Zeta regions.

Crucial interactions that are responsible for complex formation and for blocking the toxic activity of Zeta are located at the interface of the Epsilon–Zeta heterodimer, mainly between α-helix A of Epsilon and the corresponding binding groove on the surface of the Zeta subunit. Thus, disruption of the interaction between the Zeta and Epsilon subunits should be sufficient to induce cellular toxicity. The three α-helices of the Zeta protein that constitute the binding interface with Epsilon in the tetrameric complex were selected as potential peptide ligands, I (AA8-25, red), II (AA43-58, yellow), and III (AA149-165) (Figure 1 and Figure 2; Table 1), and were further evaluated.

The Zeta fragment comprising residues 67–75 (0), which is also responsible for formation of the Epsilon–Zeta interface architecture, was rationally omitted in further experimental studies, due to the fact that the corresponding surface buried on the Epsilon monomer has a very narrow character and is unsuitable for targeting by drug-like molecules in a future step of the drug development process. In order to exclude the aggregation of native Zeta-based peptides with Zeta protein, which could interfere with its folding process and/or block its toxicity [56,57], we introduced mutations into the designed peptides. All amino acids in the three helical peptides interacting with Epsilon were left unchanged, whereas in the helix interfaces interacting with other parts of the Zeta protein, two amino acids were modified in the native sequence (Table 1). Finally, to more precisely map the hot-spot areas of the Epsilon–Zeta interface, the proposed peptides were synthesized in three different lengths (Table 2).

### 2.2. Synthesis of Fluorescein- and Acetyl-Labeled Peptides

The synthesis of in silico proposed peptides was performed as described in the Section 4. Briefly, synthesis was performed by using solid phase peptide methodology, using 2-chloro-trityl resin as a polymer support. For all peptides, amino acid coupling was performed using the corresponding Fmoc-protected amino acid. Each peptide was modified by the introduction of an acetyl or 5-(6)-fluoresceinyl-carboxy group at the N-terminus, generating a focused library of fluorescent and non-fluorescent probes [49,50] (Table 2). Their ability to interact with Epsilon was evaluated experimentally by a fluorescence polarization (FP) assay. The peptides Fluo-III(a–c) and Ac-III(a–c) were obtained with oxidized methionine residues containing a sulfoxide in the side chain. Even when the synthesis was conducted under a nitrogen atmosphere and when triisopropylsilane (TIS) was added to various modified cleavage mixtures [58,59,60,61,62], quantitative formation of the sulfoxide derivatives was observed. After HPLC purification and lyophilization, the molecular weights were confirmed by mass spectrometry using the nLC-MS/MS ESI (electrospray ionization) interface (Table 2). The purity of all fluorescent peptides was over 95%, based on their HPLC results.

### 2.3. Characterization of Epsilon–Peptide Interactions by Fluorescence Polarization

**Figure 3:**
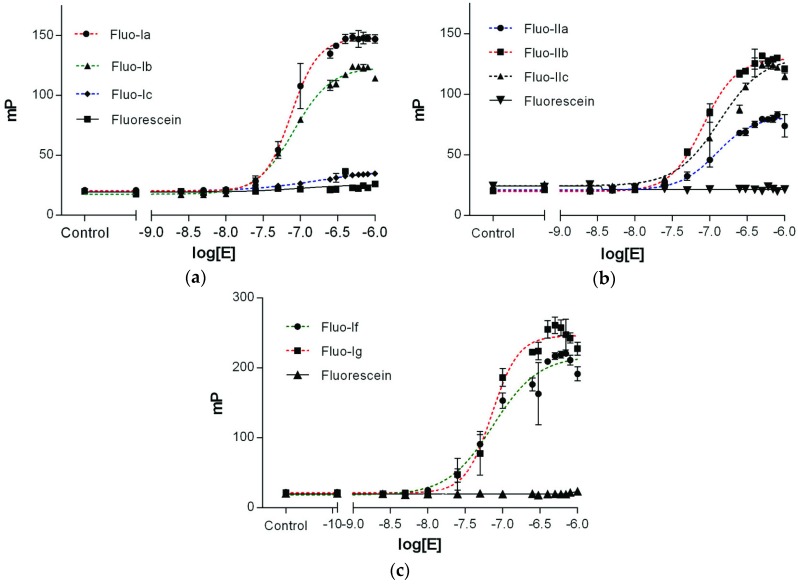
Dose-response curve for the binding of Fluo-(I–II) to the Epsilon protein. The fluorescent-labeled peptides Fluo-(I–II) at a concentration of 10 nM were treated with increasing concentrations of Epsilon (from 0.05 nM to 2.5 µM) in 10 mM HEPES assay buffer, pH 7.5, with 0.1% Tween-20. Values are plotted as the means ± standard deviation (*n* = 3) from three independent experiments. (**a**) Peptide Fluo-Ia (**●**); Fluo-Ib (▲); Fluo-Ic (♦); (**b**) Fluo-IIa (●); Fluo-IIb (■); Fluo-IIc (▲); (**c**) Fluo-If (●) and Fluo-Ig (■). Nonspecific binding of fluorescein in graphs a, b, c is plotted as black (■), (**▼**), (▲) respectively.

Binding affinities of the fluorescein-labeled peptides Fluo-(I–III) with Epsilon protein were determined in a fluorescence polarization (FP) assay [40,63,64,65]. For the purpose, 10 nM solutions of each fluorescein-labeled peptide containing different concentrations of Epsilon (from 5 nM to 7.5 µM) were irradiated with polarized light at the excitation wavelength of 470 nm and the polarization of the emitted light was recorded in mP at 520 nm (see Section 4). Binding affinities of all peptide ligands were determined by plotting the observed polarization values as a function of the logarithm of the protein concentration as *EC_50_* values (Figure 3). As in all experiments, the fluorescence intensities of the bound ligand (*I*_B_) were equal to those of the free ligand in solution (*I*_F_), i.e., the ratio *Q* = *I*_B_/*I*_F_ was close to 1 (Figure S1, Supplementary Materials); the protein concentration at the half-maximal polarization value (*EC_50_*) corresponds directly to the binding affinity *K*_D_ value of the fluorescent peptide ligand (Table 2). Fluorescent peptides Fluo-Ia, b and Fluo-II(a–c) showed binding affinities for Epsilon in the nanomolar range, whereas no saturation of the polarization with increasing protein concentrations was observed for peptides Fluo-III(a–c), derived from the α-helix G of Zeta, indicating that the latter peptides have no affinity for the Epsilon protein.

N-terminally fluorescein-labeled peptides Fluo-I(a–c) derived from the α-helix A (red in Figure 1 and Figure 2) showed higher binding affinities (i.e., lower *K*_D_ values) with shortening of the amino acid sequence. The decapeptide Fluo-Ia was most effective in binding to the Epsilon protein, with a *K*_D_ of 74.5 nM, whereas the 18mer-peptide Fluo-Ic lacked affinity toward the Epsilon protein. The peptides Fluo-II(a–c) displayed affinities in the *K*_D_ range between 99.0 and 170 nM that did not depend strongly on the peptide length with the 14-mer Fluo-IIb as the best in the series. The fluorescent peptide Fluo-Ia was selected for further development and optimization of the FP assay based on the following considerations: (i) the high binding affinity (*K*_D_ 74.5 nM), which allows the detection limit of the assay to be increased; and (ii) the large dynamic range, which will provide a better signal to noise ratio (ΔmP = 127). With this peptide, the influence of either the salt (NaCl) concentration or the DMSO proportion, a commonly solvents used in high-throughput screening, on the binding constant was studied.

The obtained results showed that the binding affinity of the fluorescent peptide Fluo-Ia is affected by the salt concentration, modifying either the dynamic range or the shape of the binding curve (Figure S2, Supplementary Materials). In general, increasing the salt concentration (up to 100 mM) drastically reduces the interaction of Fluo-Ia with the Epsilon, not considering the saturation curve. In presence of 10 mM NaCl, the binding constant is 1.4 times less than in the absence of NaCl (105 vs. 74 nM). The influence of the DMSO proportion (up to 2% DMSO) on the binding constant was studied. In this case, the binding constant, the dynamic range and the shape of the binding curve were not altered, indicating that the FP assay is stable up to 2% DMSO (Figure S2, Supplementary Materials).

**Figure 4:**
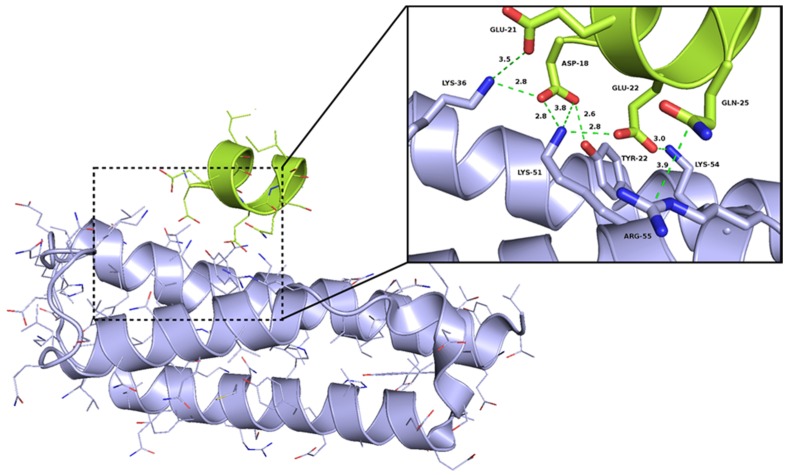
Interaction between Epsilon and the most active peptide Ia. The picture visualizes the electrostatic interactions between Epsilon and Ia—the distances between most crucial atoms (red—oxygen, blue—nitrogen) have been shown in the close-view picture by dashed lines. The Epsilon and Ia has been shown as violet and green cartoon representations, respectively.

The potential binding mode of peptide Ia was proposed by structure-based modeling. For this purpose, a binding model of Ia was derived from the crystal structure (Figure 4), suggesting that binding between the peptide and the Epsilon protein mainly occurred through hydrogen bonds and electrostatic interactions between the negatively charged residues of the peptide Ia (D18 and E22) and the complementary, positively charged residues on the surface of the Epsilon protein, namely K36, K51, K54, and R55. The nitrogen atom of Epsilon K51 is predicted to be located 2.8 and 3.8 Å away from the O1 and O2 atoms, respectively, of the D18-carboxylate in Ia. The O1 and O2 atoms of D18 in Ia could also form additional hydrogen bonds with the side chain of Epsilon K36 and the hydroxyl group of Y22, respectively, because the distance between donor and acceptor in both cases is approximately 2.8 Å. In the case of E22 from Ia, the atoms O1 and O2 of the carboxylate group are close to the Epsilon residues K51 (2.8 Å) and K54 (3.0 Å), respectively. Binding of Ia and Epsilon appears to be stabilized further by the interactions between E22 and D25 of Ia and K36 and R55 of Epsilon, respectively. Moreover, mutagenic studies have shown that the mutation D18A in the α-helix A of Zeta abolished the interaction between the Epsilon–Zeta pair [24]. In order to investigate the relevance of D18 for the interaction with Epsilon on the peptide level the D18A mutation was introduced into the Fluo-Ia furnishing peptide Fluo-Id. In addition, the aspartate D18 was exchanged with L20 obtaining Fluo-Ie (D18L, L20D with respect to Fluo-Ia). In the D18A mutation of Fluo-Ia completely abolished the binding between Fluo-Id and Epsilon (Table 2/Figure S3). In the case of the double mutation Fluo-Ie, the binding affinity in comparison to Fluo-Ia was reduced more than twofold (*K*_D_ 175 nM vs. 74.5 nM, see Table 2 and Figure S3). Shifting the aspartate residue from D18 to D20 by exchange with L20 did not abolish binding of Fluo-If to Epsilon completely and the remaining *K*_D_ value suggests that the aspartate in position D20 can still interact with the cationic cluster of the Epsilon protein. Thus, the two mutants of peptide Ia, Fluo-Id, and Fluo-Ie both confirmed the importance of the D18 amino acid residue for binding. Finally, the significance of the Zeta hot spot D18-E22 for complex formation with Epsilon was challenged by preparing the even further shortened hexa- and penta-peptides Fluo-If and Fluo-Ig, retaining the (N)DNLEE sequence but omitting the C-terminal amino acids. Both peptides Fluo-If and Fluo-Ig maintained the nanomolar affinities (74.4 ± 8.05 nM and 71.6 ± 9.15 nM, respectively) (Table 2/Figure 3) and thus represent the short binding motif of the Zeta-Epsilon interaction. It is worth mentioning, that our best obtained peptides show an affinity one order of magnitude higher than observed in the Epsilon–Zeta complex (*K*_D_ = 1 µM) [26].

### 2.4. Competitive Binding Assays

**Figure 5:**
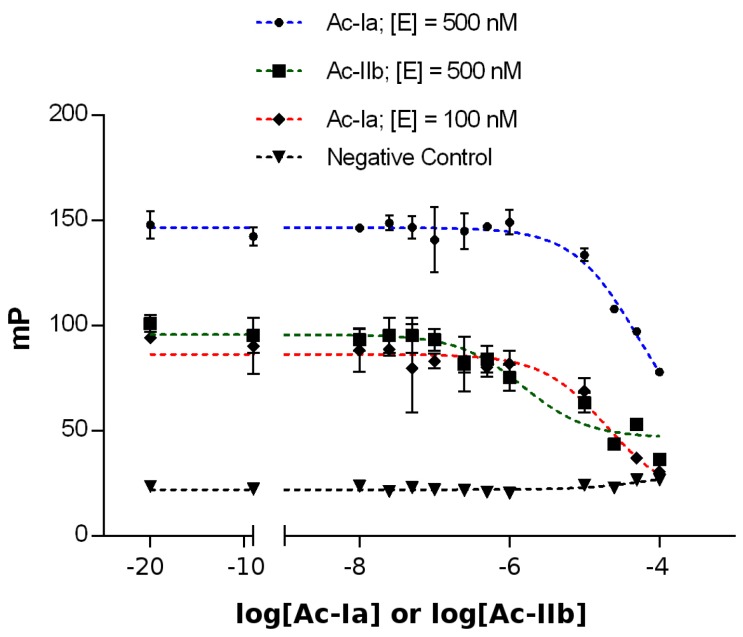
Competitive binding of the fluorescein-labeled peptides Fluo-Ia and Fluo-IIb to the Epsilon protein in 384-well plates. Epsilon (100 nM (♦) or 500 nM (●), final concentration) was incubated with 10 nM (final concentration) of Fluo-Ia and different concentrations of Ac-Ia (ranging from 0.01 to 100 µM) in 10 mM HEPES assay buffer, pH 7.5, with 0.1% Tween-20. Epsilon protein (500 nM (■), final concentration) was incubated with 10 nM (final concentration) of Fluo-IIb and different concentrations of Ac-IIb (ranging from 0.01 to 100 µM). Fluorescence polarization was measured on a SAFIRE II (Tecan, Crailsheim, Germany) microplate reader, using an excitation wavelength of 470 nm and emission wavelength of 525 nm. Values are plotted as the means of duplicates in three independent experiments ± SD. *IC_50_* values of 18.6 ± 4.64 µM (100 nM) and 51.8 ± 7.4 µM (500 nM) were obtained for the peptides Fluo-Ia and Ac-Ia, respectively.

The fluorescent high-affinity peptide ligands could be the basis for an especially economic and reliable high-throughput assay, if the fluorescence polarization read-out can be used for the screening of potential ligands that are able to bind to Epsilon in a competitive binding assay. For this purpose, we utilized the unlabeled peptides Ac-Ia and Ac-IIb containing the same amino acid sequence as the initial peptides but with their N-terminus modified by the introduction of an acetyl group [66]. The competitive binding assays were performed by using appropriate amounts of Epsilon protein based on the binding affinity, expressed in terms of the *K*_D_ value, for the labeled peptides Fluo-Ia and Fluo-IIb. In both cases, the protein concentration was set to the *K*_D_ value and 5-fold the *K*_D_ value to study the influence of the protein concentration on the *IC_50_*-value of every acetylated peptide. Thus, the Epsilon protein (100 nM or 500 nM final concentration) was treated with Fluo-Ia or Fluo-IIb (10 nM final concentration). After incubation, each mixture was treated with different concentrations of the unlabeled peptides Ac-Ia and Ac-IIb, ranging from 0.01 to 100 µM. Figure 5 shows the competitive binding curves for the labeled Fluo-Ia and Fluo-IIb peptides. Binding curves were fitted with a dose-response inhibition model (log c (inhibitors) vs. response, three parameters) and provided *IC_50_* values of 18.6 ± 4.64 µM (100 nM of Epsilon) and 51.8 ± 7.4 µM (500 nM of Epsilon) for the peptide Ac-Ia and 15.3 ± 2.5 µM (500 nM of Epsilon) for the peptide Ac-IIb, respectively. The ability to identify active compounds (hits) from a particular high-throughput screening (HTS) assay depends largely on the suitability or quality of the assay conditions used in the screening. In this context, the Z’-factor provides a useful tool for comparing and evaluating the quality of the assay described in terms of four parameters related to the mean and standard deviation of both positive and negative controls (see Supplementary Materials and Section 4). To determine if our assay system can be used to identify active compounds (hits) from large chemical libraries, the reliability of the 384-well Epsilon FP assays was tested by performing Z’-factor analysis. The expected Z’-factor values for a reliable assay should be between 0.5 and 1.0 [67], which was found for Fluo-Ia and Fluo-IIb (0.881 and 0.839, respectively) (Figure S4, Supplementary Materials).

## 3. Conclusions

In summary, the presented results have revealed a strong peptide–protein interaction within the ε_2_ζ_2_ complex that holds promise for the development of small molecule ligands able to disrupt the Epsilon-Zeta TA system. The fluorescent peptide discovered in this work enabled the direct determination of protein binding by fluorescence anisotropy measurements and bound to Epsilon with nanomolar affinity. Applying the fluorescent peptidic ligands in competitive binding assays with non-fluorescent ligands showed that this could be an excellent tool for the rapid and efficient screening of chemical libraries of drug-like molecules. Thereby, it will enable us to find inhibitors of protein–protein interactions of the Epsilon–Zeta TA system that could be effective antibacterial agents. In particular, genes predicted to encode TA systems are highly abundant in free-living bacteria but are absent from the genomes of host-associated bacteria as well as from human cells [68].

## 4. Materials and Methods

### 4.1. Peptide Design

Peptide ligands were designed by in silico analysis of a tetramer ε_2_ζ_2_ complex crystal structure [54]. First, the heterodimer ε/ζ interaction interfaces have been defined according to the method implemented in the COCOMAPS server [69,70]; the cutoff distance was set to 8 Å. The three continuous sequence fragments of Zeta, which contain interface residues, were selected as prototypes for the final peptides: 8-TDKQFENRLNDNLEELIQ-25 (I), 43-GSGKTSLRSAIFEETQ-58 (II), and 149-INSYLGTIERYETMYADD-165 (III). However, another fragment, 67-DTFKQQHPN-75 (0), was omitted in further experimental studies (for more details, see Section 2). Further modifications of the selected sequences have been proposed to disallow the potential effect of native Zeta sequences interfering with correct Zeta protein folding. Thus, following mutations have been introduced: D9R and L20H for peptide I, K46D and I53E for peptide II, and L153H and E160K for peptide III. All structural analyses and figure visualizations were conducted by using PyMOL [71] (Version 1.7, Schrödinger, LLC, New York, NY, USA).

### 4.2. Reagents and Resins

Reagents and resins were purchased from Sigma-Aldrich (Saint Louis, MO, USA), Acros Organics (Geel, Belgium), and Novabiochem (Darmstadt, Germany) and were used without further purification, unless otherwise stated. The Fmoc-protected amino acids used in this work were Fmoc-Ile-OH, Fmoc-Leu-OH, Fmoc-Glu(OtBu)-OH, Fmoc-His(Trt)-OH, Fmoc-Asn(Trt)-OH, Fmoc-Asp(OtBu)-OH, Fmoc-Arg(Pbf)-OH, Fmoc-Phe-OH, Fmoc-Gln(Trt)-OH, Fmoc-Lys(Boc)-OH, and Fmoc-Thr(tBu)-OH.

### 4.3. Synthesis

Fluorescein-labeled peptides were synthesized via Fmoc-mediated solid phase synthesis on 2-chloro-2-trytyl chloride resin (200 mg, *f* = 1.18 mmol·g^−1^). First, the resin was placed in a 10-mL polypropylene syringe fitted with a polyethylene filter disk and was treated with the corresponding Fmoc-protected amino acid (Fmoc-Gln(trt)-OH or Fmoc-Asp(OtBu)-OH; 4 eq.) and with diisopropylethylamine (DIPEA, 4 eq.) in DMF for 15 min at room temperature. Subsequently, an additional portion of DIPEA (8 eq.) was added, and the reaction mixture was stirred for 18 h at room temperature. The resin was filtered off, washed three times with DMF, CH_2_Cl_2_, and Et_2_O, and dried under a high vacuum. When the reaction was finished, the loading was calculated by UV spectroscopy (*f* = 1.18 mmol·g^−1^) (Jasco V-500 UV/Vis, Oklahoma City, OK, USA). The remaining coupling positions were capped with MeOH/DMF (0.5:1, *v*/*v*). The Fmoc group was removed by treatment with 20% piperidine in DMF twice, for 10 min. The amino acids were coupled by the addition of 4 eq. of Fmoc-protected amino acids, 4 eq. of diisopropylcarbodiimide (DIC), and 4 eq. of hydroxybenzotriazole (HOBt) in DMF for 2 h at room temperature. Fmoc deblocking and coupling steps were repeated for additional amino acid couplings. The completion of all couplings was confirmed with a negative ninhydrin test result by using Kaiser’s test [72]. After 10 amino acid couplings, the resin was split into three portions; two of the portions were used for the synthesis of modified peptides with 10 amino acid residues, and the other portion was used for forward synthesis. After the last amino acid coupling for each sequence, the Fmoc protective group was removed with 20% piperidine in DMF, and the amino terminus was acetylated or modified by the introduction of 5-(6)-carboxyfluorescein. For the acetylated peptide, the resin was treated twice with 4 eq. of acetic anhydride (Ac_2_O) for 2 h at room temperature, until the ninhydrin test was negative. Treatment of the resin with 4 eq. of 5-(6)-carboxyfluorescein, 4 eq. of HOBt, and 4 eq. of DIC in DMF for 6 h at room temperature (until the ninhydrin test was negative) afforded the corresponding fluorescein-labeled peptides. Finally, the peptides were cleaved from the resin, and the protective amino acid side chains were removed using a cleavage cocktail of TFA/TIS/H_2_O/EDT (94:1:2.5:2.5) or TFA/TMBS/TIS/EDT (92:3.5:1:3.5) for 3 h at room temperature. The filtrates were collected, and the solvents were removed under an N_2_ current. Each product was precipitated by the addition of cleavage solution to cold diethyl ether or methyl-*t*-butyl ether (4 °C). After centrifugation (4000 min^−1^, 3 min) the upper phase was removed from the vial, and the peptides were washed with diethyl ether or methyl-*t*-butyl ether for 5 min in an ultrasonic bath. Then, the vial was centrifuged. The remaining solid was dissolved in CH_3_CN/H_2_O (1:4, *v*/*v*) and lyophilized. After that, each peptide was purified by reverse HPLC (Agilent Technologies 1260 Infinity equipment, Santa Clara, CA, USA) using a reverse phase column (Nucleodur, C-18 HTec, 5 µm, Macherey-Nagel, Düren, Germany), and a solvent mixture of water and acetonitrile containing 0.01% TFA, starting from 5% B to 95% B over 40 min, at a flow rate 30 mL·min^−1^ with a linear gradient). The final peptides were isolated as a white or yellow solid after lyophilization, yielding between 10% and 15%. LC-MS analyses were conducted on an Agilent 1100 system equipped with a reverse phase column (Polaris-NH_2_, C-8 column, 3 µm, L × ID = 100 × 4.6 mm, Agilent Technologies, Santa Clara, CA, USA) operated with acetonitrile-water mixtures containing 0.1% formic acid, a diode array detector and a single quadruple mass spectrometer with electrospray ionization (ESI). For nLC-MS/MS analysis, the synthetic peptides were dissolved in a solution containing 2.5% acetonitrile (ACN, HPLC grade, Carl Roth GmbH) and 0.1% formic acid (HCOOH). The employed nLC-MS/MS system comprises an 1100 series nanoHPLC dual pump system (Agilent Technologies, Santa Clara, CA, USA) and a LTQ-FT mass spectrometer (Thermo Fisher Scientific, San José, CA, USA). A Nanomate ESI (electrospray ionization) interface (Advion) was used to couple the separation and the MS techniques. For sample desalting and pre-concentration, the injected sample was loaded on a trap column (Zorbax 300SB C18, 5 µm, 5 mm × 0.3 mm) at a flow rate of 25 µL·min^−1^, using 5% ACN and 0.1% HCOOH as the mobile phase, for 5 min. Then, preconcentrated peptides were eluted and transferred to a reverse phase nanocapillary analytical column (Zorbax 300SB C18, 3.5 µm pore size, 150 mm × 75 µm) by reversing the flow at 350 nL·min^−1^. Peptide elution was performed applying a two-step gradient: 10%–50% B linear in 33 min, 50%–90% B linear in 3 min, holding the system at 90% B for 4 min. Mobile phases used were: A, 5% ACN, 0.1% HCOOH, and B, 99.9% ACN, 0.1% HCOOH. During the MS analysis, the instrumental parameters were set as follows: spray voltage 1.7 kV (positive mode); capillary temperature 200 °C. High resolution full scan spectra were acquired in the FT analyzer for exact mass identification (*m*/*z* range 350–2000, resolution 100,000). Alternatively, collision induced dissociation (CID) MS/MS fragmentations (35% normalized collision energy, 4 Da isolation window) were detected in the ion trap to confirm the identity.

### 4.4. Protein Purification

The *N*-terminally hexa-histidine-tagged His6-Epsilon protein was overproduced in *E. coli* BL21 (DE3) and purified under native conditions, as previously described [37]. Purified protein eluted from a Ni-TED column was dialyzed against the storage buffer (25 mM Tris pH 8, 100 mM KCl, 5 mM DTT, 1 mM MgCl_2_, 50% glycerol) and then stored at −20 °C. Before being used in FP assays, the His6-Epsilon protein was dialyzed against the storage buffer without glycerol. After dialysis, the His6-Epsilon protein sample was centrifuged at 4 °C at 7000× *g* for 3 min to remove the insoluble fraction, concentrated using the AmiconUltra-4 10K filter device (Millipore, Billerica, MA, USA) and stored at 4 °C. The purity of the protein was verified by SDS-PAGE with Coomassie blue staining. The protein concentration was determined by comparing the intensity of the protein bands with that of the bovine serum albumin standard using Multi Gauge V3.0 software (Fujifilm, Tokyo, Japan). The quality and identity of the protein was also determined by Matrix-Assisted Laser Desorption/Ionization Mass Spectrometry (MALDI)-TOF, using sinapic acid as a matrix (Figure S5, Supplementary Materials).

### 4.5. Fluorescence Polarization Assay

For fluorescence polarization measurements, peptides were dissolved in DMSO at 10 mM and diluted in 10 mM HEPES (pH 7.5) containing 0.1% Tween-20. The assay was conducted on untreated black 384-well microtiter plates (low volume, non-binding surface, round bottom, non-sterile black polystyrene, Corning B.V. Life Sciences, Nr. 3676, Kennebunk, ME, USA) in a final volume of 10 µL, using 10 mM HEPES (pH 7.5) with 0.1% Tween-20 as a buffer. The final DMSO proportion in the assay was less than 0.1%. The measurements were performed on a SAFIRE II (Tecan) microplate reader, using an excitation wavelength of 470 nm and emission wavelength of 525 nm. The final assay mixtures contained 5 µL fluorescein-labeled peptide (furnishing a final assay concentration of 10 nM), and 5 µL of increasing amounts of Epsilon protein (final concentrations ranging from 0.05 nM to 5 µM). The negative controls comprised 5 µL fluorescein-labeled peptide (maintaining a final assay concentration of 10 nM) and 5 µL 10 mM HEPES buffer (pH 7.5) containing 0.1% Tween-20. To avoid protein aggregation, Tween-20 (0.1% final concentration) was used as a detergent in all experiments. After adding the fluorescent peptide, the plates were centrifuged (2000 min^−1^, 3 min) and shaken briefly (2000 rpm for 10 min at room temperature), and polarization of the emitted fluorescence was directly recorded. In every case, the well containing only the fluorescein-labeled peptide was subtracted from the assay well as background. The polarization values (mP) were plotted as a function of the logarithm of the protein concentration. Binding curves were fitted by using GraphPad Prism software (version 4 for Windows, GraphPad, San Diego, CA, USA) employing a sigmoidal dose-response model (4 PL), and *K*_D_ values in the nanomolar range were obtained. All of the experiments were carried out in duplicate in three independent experiments, and the results are expressed as the mean ± standard deviation (SD).

### 4.6. Competitive Binding Assays

For the competitive binding assays, unlabeled peptides containing the same amino acid sequence as the previous one but with the N-terminal end modified by the introduction of acetyl group were used. In this assay, the final Epsilon protein concentration was set at the *K*_D_ concentration defined for each peptide. The assay was conducted on untreated black 384-well microtiter plates (low volume, non-binding surface, round bottom, non-sterile black polystyrene, Corning B.V. Life Sciences, Nr. 3676, Kennebunk, ME, USA) in a final volume of 10 µL, using 10 mM HEPES (pH 7.5) with 0.1% Tween-20 as a buffer. The final DMSO proportion in the assay was less than 1%. The measurements were performed on a SAFIRE II microplate reader (Tecan), using an excitation wavelength of 470 nm and emission wavelength of 525 nm. The final assay mixtures comprised 1 µL fluorescein-labeled peptide (maintaining a final assay concentration of 10 nM), and 5 µL of the corresponding Epsilon concentration and 4 µL of a different concentration of unlabeled peptide ranging from 0.01 to 100 µM. To avoid protein aggregation, Tween-20 (0.1% final concentration) was used as a detergent in all experiments. After addition of the fluorescent peptide and the protein, the plates were centrifuged (2000 min^−1^, 3 min) and shaken briefly (2000 rpm for 10 min at room temperature), and the mixture was incubated for 10 min at room temperature. Then, 4 µL of the acetyl-labeled peptide was added (final concentration ranging from 0.01 µM to 100 µM). The mixtures were centrifuged (2000 min^−1^, 3 min) and shaken briefly (2000 rpm for 10 min at room temperature), and the polarization of the emitted fluorescence was directly recorded. In every case, the negative controls formed by a mixture of the fluorescein-labeled peptide plus acetyl-labeled peptides were subtracted from the assay well as background. The polarization values (mP) were plotted as a function of the logarithm of the protein concentration. Binding curves were fitted by using GraphPad Prism software (version 4 for Windows GraphPad, La Jolla, CA, USA) with a sigmoidal dose-response model (4 PL) and provided *IC_50_* values in the micromolar and submicromolar ranges. All of the experiments were carried out in duplicate in three independent experiments, and the results are expressed as the mean ± standard deviation (SD).

### 4.7. Calculation of the Z’-Factor

This value is used to compare a large number of compounds from a single measurement of an unknown sample to positive and negative control samples. For Z’-factor determination, the assay was carried out on untreated black 384-well microtiter plates (low volume, non-binding surface, round bottom, non-sterile black polystyrene, Corning B.V. Life Sciences, Nr. 3676, Kennebunk, ME, USA) in a final volume of 10 µL, using10 mM HEPES (pH 7.5) with 0.1% Tween-20 as a buffer. The final assay mixtures were composed as follows: (a) in a bound state (positive control), by using a mixture of 5 µL fluorescein-labeled peptide (maintaining a final assay concentration of 10 nM) and 5 µL of Epsilon protein (final concentration of 500 nM or 750 nM, depending on the fluorescein-labeled peptide); and (b) in a free state (negative control), by using a mixture of 5 µL buffer, 5 µL fluorescein-labeled peptide (maintaining its final assay concentration at 10 nM). After that, the plates were centrifuged (2000 min^−1^, 3 min) and shaken briefly (2000 rpm for 10 min at room temperature), and polarization of the emitted fluorescence was directly recorded at an emission wavelength of 525 nm. The mean and standard deviation from the bound and free states were calculated to subsequently determine the Z’ value using Equation (1):

(1) $$Z^{'}-\text{factor}=1-\frac{\left(\sigma_{p}+\sigma_{n}\right)}{\left(\mu_{p}-\mu_{n}\right)},$$

where the Z’-factor is defined in terms of four parameters: the means and standard deviations of both the positive (*p*) and negative (*n*) controls (µ*_p_*, σ*_p_*, µ*_n_*, σ*_n_*).

## Figures and Tables

**Table 1:** Description of the native amino acid sequences of Zeta protein and their corresponding unnatural modification considered in this study. The amino acids that have been changed in the original amino acid sequence are in bold.

Peptide Abbreviations	Peptide Sequences
I (native)	8-TDKQFENRLNDNLEELIQ-25
Ia	18-LNDN**H**EELIQ-25
Ib	12-FENRLNDN**H**EELIQ-25
Ic	8-T**R**KQFENRLNDN**H**EELIQ-25
II (native)	43-GSGKTSLRSAIFEETQ-58
IIa	49-LRSA**E**FEETQ-58
IIb	45-G**D**TSLRSA**E**FEETQ-58
IIc	43-GSG**D**TSLRSA**E**FEETQ-58
III (native)	149-INSYLGTIERYETMYADD-166
IIIa	157-ERY**K**TMYADD-166
IIIb	153-**H**GTIERY**K**TMYADD-166
IIIc	149-INSY**H**GTIERY**K**TMYADD-166

**Table 2:** Amino acid sequence of the proposed fluorescein-labeled peptides Fluo-I-III, their acetylated homologs, Ac-I-III, and binding constant determination for the fluorescein-labeled.

Entry	Peptide	Peptide Sequence	tR ^a^ (min)	Mass ^d^		Binding Constant Values	
				Calculated	Found	*K*_D_ (nM) ^e^	ΔmP
1	Fluo-Ia	Fluo-LNDNHEELIQ	19.7 ^b^	1582.6332	1582.6327	74.5 ± 10.5	127
2	Fluo-Ib	Fluo-FENRLNDNHEELIQ	17.4 ^b^	2128.8883	2128.8901	89.5 ± 13.9	105
3	Fluo-Ic	Fluo-TRKQFENRLNDNHEELIQ	17.2 ^b^	2642.1906	2642.1822	No binding	-
4	Fluo-IIa	Fluo-LRSAEFEETQ	20.1 ^b^	1567.6223	1567.6235	169.5 ± 16.0	61
5	Fluo-IIb	Fluo-GDTSLRSAEFEETQ	18.1 ^b^	1927.7505	1927.7498	99.5 ± 12.2	110
6	Fluo-IIc	Fluo-GSGDTSLRSAEFEETQ	18.3 ^b^	2071.8040	2071.8009	134.0 ± 15.0	106
7	Fluo-IIIa	Fluo-ERYKTMYADD	17.4 ^b^	1649.6213	1650.2056	No binding	-
8	Fluo-IIIb	Fluo-HGTIERYKTMYADD	16.2 ^b^	2057.8334	2057.0183	No binding	-
9	Fluo-IIIc	Fluo-INSYHGTIERYKTMYADD	18.4 ^b^	2536.0398	2536.1358	No binding	-
10	Ac-Ia	Ac-LNDNHEELIQ	14.3 ^c^	1266.5961	1266.5998	n/a	n/a
11	Ac-Ib	Ac-FENRLNDNHEELIQ	14.0 ^c^	1812.8511	1812.8543	n/a	n/a
12	Ac-Ic	Ac-TRKQFENRLNDNHEELIQ	13.2 ^c^	2325.1682	2325.1653	n/a	n/a
13	Ac-IIa	Ac-LRSAEFEETQ	15.8 ^c^	1251.5852	1251.5811	n/a	n/a
14	Ac-IIb	Ac-GDTSLRSAEFEETQ	13.8 ^c^	1611.7133	1611.7113	n/a	n/a
15	Ac-IIc	Ac-GSGDTSLRSAEFEETQ	13.5 ^c^	1755.7668	1755.7641	n/a	n/a
16	Ac-IIIa	Ac-ERYKTMYADD	13.4 ^c^	1333.5841	1333.5891	n/a	n/a
17	Fluo-Id	Fluo- LNANHEELIQ	18.5 ^c^	1538.6434	1538.6474	No binding	-
18	Fluo-Ie	Fluo-DNLNHEELIQ	18.3 ^c^	1582.6332	1582.6327	175.0 ± 8.90	115
19	Fluo-If	Fluo-NDNLEE	17.5 ^c^	1091.3476	1091.3465	74.4 ± 8.05	199
20	Fluo-Ig	Fluo-DNLEE	17.5 ^c^	976.2974	976.2969	71.6 ± 9.15	225

^a^ HPLC purification using Agilent Technologies (Santa Clara, CA, USA) 1260 Infinity equipment, a reverse phase column (Nucleodur, C-18 HTec, 5 µm, Macherey-Nagel, Düren, Germany), and a solvent mixture of water and acetonitrile containing 0.01% TFA. The purity % (UV, λ = 210 nm), for all peptides was 100%; ^b^ Purification was performed starting from 25% B to 95% B over 40 min at a flow rate of 30 mL·min^−1^ with a linear gradient; ^c^ Purification was performed starting from 5% B to 95% B over 40 min at a flow rate of 30 mL·min^−1^ with a linear gradient; ^d^ nLC-MS/MS ESI (electrospray ionization) interface (Advion). Fluo: *N*-5-(6)-fluoresceinyl-carboxy-; Ac: acetyl group; ^e^ Values represent three independent experiments, including three replicates (*n* = 3 ± SD); n/a: not applicable.

## Supplementary Materials

The following are available online at www.mdpi.com/2072-6651/8/7/222/s1.
