# Supplementary material for: Mapping Protein–Protein Interactions of the Resistance-Related Bacterial Zeta Toxin–Epsilon Antitoxin Complex (ε2ζ2) with High Affinity Peptide Ligands Using Fluorescence Polarization
Source: Toxins (Basel). 2016 Jul 16;8(7):222. doi: 10.3390/toxins8070222 (PMC4963854; doi:10.3390/toxins8070222)
Supplement: Supplementary file 1 [file toxins-08-00222-s001.pdf]

Supplementary Materials: Mapping Protein-Protein Interactions of the Resistance-Related Bacterial Zeta Toxin–Epsilon Antitoxin Complex ($\varepsilon_2\zeta_2$) with High Affinity Peptide Ligands Using Fluorescence Polarization

María Isabel Fernández-Bachiller, Iwona Brzozowska, Norbert Odolczyk, Urszula Zielenkiewicz, Piotr Zielenkiewicz and Jörg Rademann

1. Solid-phase peptide synthesis

1.1. Fluo-I(a–c) and Ac-I(a–c)

For each peptide synthesis, 2-chloro-2-trityl chloride resin (200 mg, f = 1.18 mmol·g^{-1}) placed in a 10 mL polypropylene syringe fitted with a polyethylene filter disk was treated with Fmoc-Gln(trt)-OH (410 mg, 0.67 mmol, 4 equiv.) and with diisopropylethylamine (DIPEA; 117 µL, 0.67 mmol, 4 equiv.) in DMF for 15 min at room temperature. Subsequently, an additional portion of DIPEA (235 µL, 1.52 mmol, 8 equiv.) was added and the reaction mixture was stirred for 18 h at room temperature. The resin was filtered off, washed three times with DMF, CH_2Cl_2 and Et_2O, and dried under high vacuum. When the reaction was finished, the loading was calculated by UV spectroscopy (f = 0.88 mmol·g^{-1}). The chain was elongated by sequentially coupling Fmoc-Ile-OH, Fmoc-Leu-OH, Fmoc-Glu(O*t*Bu)-OH, Fmoc-Glu(O*t*Bu)-OH, Fmoc-His(trt)-OH, Fmoc-Asn(trt)-OH, Fmoc-Asp(O*t*Bu)-OH, Fmoc-Asn(trt)-OH, Fmoc-Leu-OH, Fmoc-Arg(Pbf)-OH, Fmoc-Asn(trt)-OH, Fmoc-Glu(O*t*Bu)-OH, Fmoc-Phe-OH, Fmoc-Gln(trt)-OH, Fmoc-Lys(Boc)-OH, Fmoc-Arg(Pbf)-OH, Fmoc-Thr(*t*Bu)-OH by using HOBt and DIC as a coupling reagents (fourfold molar excess) in DMF for 2 h at room temperature. The completion of all couplings was confirmed with a negative ninhydrin test result. After 10 amino acid couplings the resin was spliced three portions, two of them were used for the synthesis of the peptide with 10 amino acid residues and the other portion was used for forwards synthesis. After the last amino acid coupling for each sequence, the Fmoc protective group was removed with 20% piperidine in DMF and the amino terminus was acetylated or modified by the introduction of 5-(6) carboxyfluorescein. For acetylated peptide, the resin was treated with Ac_2O (4 equiv.; 2 times until the ninhydrin test was negative) for 2 h at room temperature. The treatment of the resin with 5-(6)-carboxyfluorescein (4 equiv.), HOBt (4 equiv.), and DIC (4 equiv.) in DMF for 6 h at room temperature (until the ninhydrin test was negative) afforded the corresponding fluorescein-labeled peptides. Finally, the peptides were cleaved from the resin and the protective groups at amino acid side chains were removed with a cocktail TFA/TIS/H_2O/EDT (94:1:2.5:2.5) for 3 h at room temperature. The filtrated were collected and the solvents were removed under N_2 current. Each product was precipitated by addition of the cleavage solution to cold Et_2O or methyl *t*-butyl ether (4 °C). After centrifugation (4000 min^{-1}, 3 min) the upper phase was removed from the vial, diethyl ether or methyl-*t*-butyl ether was added, the solid was washed for 5 min in an ultrasonic bath, and the vial was centrifuged. The remaining solid was dissolved in CH_3CN/H_2O (1:4, *v*/*v*) and lyophilized. After that, each peptide was purified by reversed phase HPLC and isolated as a white solid after lyophilization in yield between 10%–15%.

Fluo-Ia (Fluorescein-LNDNHEELIQ): HPLC purity: 100% (UV, t_R = 19.7 min). ESI-MS (electrospray ionization, positive mode): *m*/*z* calcd for $C_{72}H_{92}N_{15}O_{26}$: 1582.6332 $[M + H]^+$; found: 1582.6327.

Fluo-Ib (Fluorescein-FENRLNDNHEELIQ): HPLC purity: 100% (UV, t_R = 17.4 min). ESI-MS (electrospray ionization, positive mode): *m*/*z* calcd for $C_{96}H_{126}N_{23}O_{33}$: 2128.8883 $[M + H]^+$; found: 2128.8901.

Fluo-Ic (Fluorescein-TRKQFENRLNDNHEELIQ): HPLC purity: 100% (UV, t_R = 17.2 min). ESI-MS (electrospray ionization, positive mode): *m*/*z* calcd for $C_{117}H_{165}N_{32}O_{39}$: 2642.1906 [M + H]$^+$; found: 2642.1822.

Ac-Ia (Ac-LNDNHEELIQ): HPLC purity: 100% (UV, t_R = 14.3 min). ESI-MS (electrospray ionization, positive mode): *m*/*z* calcd for $C_{53}H_{84}N_{15}O_{21}$: 1266.5961 [M + H]$^+$; found: 1266.5998.

Ac-Ib (Ac-FENRLNDNHEELIQ): HPLC purity: 100% (UV, t_R = 15.9 min). HR MS (electrospray ionization, positive mode): *m*/*z* calcd for $C_{77}H_{118}N_{23}O_{28}$: 1812.8511 [M + H]$^+$; found: 1812.8543.

Ac-Ic (Ac-TRKQFENRLNDNHEELIQ): HPLC purity: 100% (UV, t_R = 13.2 min). ESI-MS (electrospray ionization, positive mode): *m*/*z* calcd for $C_{99}H_{158}N_{31}O_{34}$: 2325.1682 [M + H]$^+$; found: 2325.1653.

1.2. Fluo-II(a–c) and Ac-II(a–c)

For each peptide synthesis, 2-chloro-2-trityl chloride resin (200 mg, *f* = 1.18 mmol·g^{-1}) placed in a 10 mL polypropylene syringe fitted with a polyethylene filter disk was treated with Fmoc-Gln(trt)-OH (410 mg, 0.67 mmol, 4 equiv.) and with diisopropylethylamine (DIPEA; 117 μL, 0.67 mmol, 4 equiv.) in DMF for 15 min at room temperature. Subsequently, an additional portion of DIPEA (235 μL, 1.52 mmol, 8 equiv.) was added and the reaction mixture was stirred for 18 h at room temperature. The resin was filtered off, washed three times with DMF, CH_2Cl_2 and Et_2O, and dried under high vacuum. When the reaction was finished, the loading was calculated by UV spectroscopy (*f* = 0.83 mmol·g^{-1}). The chain was elongated by sequentially coupling Fmoc-Thr(*t*Bu)-OH, Fmoc-Glu(O*t*Bu)-OH, Fmoc-Glu(O*t*Bu)-OH, Fmoc-Phe-OH, Fmoc-Glu(O*t*Bu)-OH, Fmoc-Ala-OH, Fmoc-Ser(*t*OBu)-OH, Fmoc-Arg(Pfb)-OH, Fmoc-Leu-OH, Fmoc-Ser(*t*OBu)-OH, Fmoc-Thr(*t*Bu)-OH, Fmoc-Asp(O*t*Bu), Fmoc-Gly-OH, Fmoc-Ser(O*t*Bu)-OH, Fmoc-Gly-OH by using HOBt and DIC as coupling reagents (fourfold molar excess) in DMF for 2 h at room temperature. The completion of all couplings was confirmed with a negative ninhydrin test result. After the last amino acid coupling for each sequence, the Fmoc protective group was removed with 20% piperidine in DMF and the amino terminus was acetylated or modified by the introduction of 5-(6)-carboxyfluorescein. For acetylated peptide, the resin was treated with Ac_2O (4 equiv.; 2 times until the ninhydrin test was negative) for 2 h at room temperature. The treatment of the resin with 5-(6)-carboxyfluorescein (4 equiv.), HOBt (4 equiv.), and DIC (4 equiv.) in DMF for 6 h at room temperature (until the ninhydrin test was negative) afforded the corresponding fluorescein-labeled peptides. Finally, the peptides were cleaved from the resin and the protective groups at amino acid side chains were removed with a cocktail TFA/TIS/H_2O/EDT (94:1:2.5:2.5) for 3 h at room temperature. The filtrated were collected and the solvents were removed under N_2 current. Each product was precipitated by addition of the cleavage solution to cold Et_2O or methyl *t*-butyl ether (4 °C). After centrifugation (4000 min^{-1}, 3 min) the upper phase was removed from the vial, diethyl ether or methyl-*t*-butyl ether was added, the solid was washed for 5 min in an ultrasonic bath, and the vial was centrifuged. The remaining solid was dissolved in ACN/H_2O (1:4, *v*/*v*) and lyophilized. After that, each peptide was purified by reversed phase HPLC (solvent A and solvent B, from 5% B to 95% B over 40 min, at flow 30 mL·min^{-1} with linear gradient) and isolated as a white solid after lyophilization in yield between 10%–15%.

Fluo-IIa (Fluorescein-LRSAEFEETQ): HPLC purity: 100% (UV, t_R = 20.1 min). ESI-MS (electrospray ionization, positive mode): *m*/*z* calcd for $C_{72}H_{91}N_{14}O_{26}$: 1567.6223 [M + H]$^+$; found: 1567.6235.

Fluo-IIb (Fluorescein-GDTSLRSAEFEETQ): HPLC purity: 100% (UV, t_R = 18.1 min). ESI-MS (electrospray ionization, positive mode): *m*/*z* calcd for $C_{85}H_{111}N_{18}O_{34}$: 1927.7505 [M + H]$^+$; found: 1927.7498.

Fluo-IIc (Fluorescein-GSGDTSLRSAEFEETQ): HPLC purity: 100% (UV, t_R = 18.3 min). ESI-MS (electrospray ionization, positive mode): *m*/*z* calcd for $C_{90}H_{119}N_{20}O_{37}$: 2071.8040 [M + H]$^+$; found: 2071.8009.

Ac-IIa (Ac-LRSAEFEETQ): HPLC purity: 100% (UV, t_R = 15.8 min). ESI-MS (electrospray ionization, positive mode): *m*/*z* calcd for $C_{53}H_{83}N_{14}O_{21}$: 1251.5852 [M + H]$^+$; found: 1251.5811.

Ac-IIb (Ac-GDTSLRSAEFEETQ): HPLC purity: 100% (UV, t_R = 14.5 min). ESI-MS (electrospray ionization, positive mode): *m*/*z* calcd for $C_{66}H_{103}N_{18}O_{29}$: 1611.7133 [M + H]$^+$; found: 1611.7113.

Ac-IIc (Ac-GSGDTSLRSAEFEETQ): HPLC purity: 100% (UV, t_R = 13.8 min). ESI-MS (electrospray ionization, positive mode): *m*/*z* calcd for $C_{71}H_{111}N_{20}O_{32}$: 1755.7668 [M + H]$^+$; found: 1755.7641.

1.3. Fluo-III(a–c) and Ac-III(a–c)

For each peptide synthesis, 2-chloro-2-trityl chloride resin (200 mg, f = 1.18 mmol·g^{-1}) placed in a 10 mL polypropylene syringe fitted with a polyethylene filter disk was treated with Fmoc-Asp(O*t*Bu)-OH (410 mg, 0.67 mmol, 4 equiv.) and with diisopropylethylamine (DIPEA; 117 µL, 0.67 mmol, 4 equiv.) in DMF for 15 min at room temperature. Subsequently, an additional portion of DIPEA (235 µL, 1.52 mmol, 8 equiv.) was added and the reaction mixture was stirred for 18 h at room temperature. The resin was filtered off, washed three times with DMF, CH_2Cl_2 and Et_2O, and dried under high vacuum. When the reaction was finished, the loading was calculated by UV spectroscopy (f = 0.75 mmol·g^{-1}). The chain was elongated by sequentially coupling Fmoc-Asp(O*t*Bu)-OH, Fmoc-Ala-OH, Fmoc-Tyr(*t*Bu)-OH, Fmoc-Met-OH, Fmoc-Thr(*t*Bu)-OH, Fmoc-Lys(BOC),-OH, Fmoc-Tyr(*t*Bu)-OH, Fmoc-Arg(Pbf)-OH, Fmoc-Glu(O*t*Bu)-OH, Fmoc-Ile-OH, Fmoc-Thr(*t*Bu)-OH, Fmoc-Gly-OH, Fmoc-His(trt)-OH, Fmoc-Tyr(*t*Bu)-OH, Fmoc-Ser(*t*Bu)-OH, Fmoc-Asn(trt)-OH, Fmoc-Ile-OH by using HOBt and DIC as a coupling reagents (fourfold molar excess) in DMF for 2 h at room temperature. The completion of all couplings was confirmed with a negative ninhydrin test result. After the last amino acid coupling for each sequence, the Fmoc protective group was removed with 20% piperidine in DMF and the amino terminus was acetylated or modified by the introduction of 5-(6)-carboxyfluorescein. For acetylated peptide, the resin was treated with Ac_2O (4 equiv.; 2 times until the ninhydrin test was negative) for 2 h at room temperature. The treatment of the resin with 5-(6)-carboxyfluorescein (4 equiv.), HOBt (4 equiv.), and DIC (4 equiv.) in DMF for 6 h at room temperature (until the ninhydrin test was negative) afforded the corresponding fluorescein-labeled peptides. After each coupling, the completion of the reaction was monitored with the Kaiser´s test, and the coupling was repeated if necessary. Due to the presence of methione in the sequence the cleavage of the peptide from the resin was carried out using a mixture of TFA/TMBS/TIS/EDT (92:3.5:1:3.5) solution for 60 min at room temperature. The filtrated were collected and the solvents were removed under N_2 current. Each product was precipitated by addition of the cleavage solution to cool methyl *t*-butyl ether (4 °C). After centrifugation (4000 min^{-1}, 3 min) the upper phase was removed from the vial, and methyl-*t*-butyl ether was added, the solid was washed for 5 min in an ultrasonic bath, and the vial was centrifuged. The remaining solid was dissolved in ACN/H_2O (1:4, *v*/*v*) and lyophilized. After that, each peptide was purified by reversed phase HPLC (solvent A and solvent B, from 5% B to 95% B over 40 min, at flow 30 mL·min^{-1} with linear gradient) and isolated as a white solid after lyophilization in yield between 10%–15%.

Fluo-IIIa (Fluorescein-ERYKTMYADD): HPLC purity: 100% (UV, t_R = 17.4 min). ESI-MS (electrospray ionization, positive mode): *m*/*z* calcd for $C_{75}H_{93}N_{16}O_{25}S$: 1649.6213 [M + H]$^+$; found: 1650.2056.

Fluo-IIIb (Fluorescein-HGTIERYKTMYADD): HPLC purity: 100% (UV, t_R = 16.2 min). ESI-MS (electrospray ionization, positive mode): *m*/*z* calcd for $C_{93}H_{121}N_{22}O_{30}S$: 2057.8334 [M + H]$^+$; found: 2057.0183.

Fluo-IIIc (Fluorescein-INSYHGTIERYKTMYADD): HPLC purity: 100% (UV, t_R = 18.4 min). ESI-MS (electrospray ionization, positive mode): *m*/*z* calcd for $C_{115}H_{151}N_{26}O_{38}S$: 2536.0398 [M + H]$^+$; found:2536.1358.

Ac-IIIa (Ac-ERYKTMYADD): HPLC purity: 100% (UV, t_R = 13.4 min). ESI-MS (electrospray ionization, positive mode): *m*/*z* calcd for $C_{56}H_{85}N_{16}O_{20}S$: 1333.5841 [M + H]$^+$; found: 1333.5891.

Ac-IIIb (Ac-HGTIERYKTMYADD): HPLC purity: 100% (UV, t_R = 14.4 min). ESI-MS (electrospray ionization, positive mode): *m*/*z* calcd for $C_{74}H_{113}N_{22}O_{25}S$: 1741.7962 [M + H]$^+$; found: 1741.7975.
Ac-IIIc (Ac-INSYHGTIERYKTMYADD): HPLC purity: 100% (UV, t_R = 14.6 min). ESI-MS (electrospray ionization, positive mode): *m*/*z* calcd for $C_{96}H_{143}N_{26}O_{33}S$: 2220.0026 [M + H]$^+$; found: 2220.0040.

1.4. Fluo-Id (Fluorescein-LNANHEELIQ)

2-chloro-2-trityl chloride resin (200 mg, *f* = 1.18 mmol·g^{-1}) placed in a 10 mL polypropylene syringe fitted with a polyethylene filter disk was treated with Fmoc-Gln(trt)-OH (410 mg, 0.67 mmol, 4 equiv.) and with diisopropylethylamine (DIPEA; 117 µL, 0.67 mmol, 4 equiv.) in DMF for 15 min at room temperature. Subsequently, an additional portion of DIPEA (235 µL, 1.52 mmol, 8 equiv.) was added and the reaction mixture was stirred for 18 h at room temperature. The resin was filtered off, washed three times with DMF, CH_2Cl_2 and Et_2O, and dried under high vacuum. When the reaction was finished, the loading was calculated by UV spectroscopy (*f* = 0.73 mmol·g^{-1}). The chain was elongated by sequentially coupling Fmoc-Ile-OH, Fmoc-Leu-OH, Fmoc-Glu(O*t*Bu)-OH, Fmoc-Glu(O*t*Bu)-OH, Fmoc-His(trt)-OH, Fmoc-Asn(trt)-OH, Fmoc-Ala-OH, Fmoc-Asn(trt)-OH, Fmoc-Leu-OH, by using HOBt and DIC as a coupling reagents (fourfold molar excess) in DMF for 2 h at room temperature. The completion of all couplings was confirmed with a negative ninhydrin test result. After 10 amino acid couplings the resin was spliced three portions, two of them were used for the synthesis of the peptide with 10 amino acid residues and the other portion was used for forwards synthesis. After the last amino acid coupling for each sequence, the Fmoc protective group was removed with 20% piperidine in DMF and the amino terminus was acetylated or modified by the introduction of 5-(6)-carboxyfluorescein. For acetylated peptide, the resin was treated with Ac_2O (4 equiv.; 2 times until the ninhydrin test was negative) for 2 h at room temperature. The treatment of the resin with 5-(6)-carboxyfluorescein (4 equiv.), HOBt (4 equiv.), and DIC (4 equiv.) in DMF for 6 h at room temperature (until the ninhydrin test was negative) afforded the corresponding fluorescein-labeled peptides. Finally, the peptides were cleaved from the resin and the protective groups at amino acid side chains were removed with a cocktail TFA/TIS/H_2O/EDT (94:1:2.5:2.5) for 3 h at room temperature. The filtrated were collected and the solvents were removed under N_2 current. Each product was precipitated by addition of the cleavage solution to cold Et_2O or methyl *t*-butyl ether (4 °C). After centrifugation (4000 min^{-1}, 3 min) the upper phase was removed from the vial, diethyl ether or methyl-*t*-butyl ether was added, the solid was washed for 5 min in an ultrasonic bath, and the vial was centrifuged. The remaining solid was dissolved in CH_3CN/H_20 (1:4, *v*/*v*) and lyophilized. After that, each peptide was purified by reversed phase HPLC and isolated as a white solid after lyophilization in yield 65%.

Fluo-Id (Fluorescein-LNANHEELIQ): HPLC purity: 100% (UV, t_R = 18.5 min). ESI-MS (electrospray ionization, positive mode): *m*/*z* calcd for $C_{71}H_{92}N_{15}O_{24}$: 1538.6434 [M + H]$^+$; found: 1538.6474.

1.5. Fluo-Ie (Fluorescein-DNLNHEELIQ)

2-Chloro-2-trityl chloride resin (200 mg, *f* = 1.18 mmol·g^{-1}) placed in a 10 mL polypropylene syringe fitted with a polyethylene filter disk was treated with Fmoc-Gln(trt)-OH (410 mg, 0.67 mmol, 4 equiv.) and with diisopropylethylamine (DIPEA; 117 µL, 0.67 mmol, 4 equiv.) in DMF for 15 min at room temperature. Subsequently, an additional portion of DIPEA (235 µL, 1.52 mmol, 8 equiv.) was added and the reaction mixture was stirred for 18 h at room temperature. The resin was filtered off, washed three times with DMF, CH_2Cl_2 and Et_2O, and dried under high vacuum. When the reaction was finished, the loading was calculated by UV spectroscopy (*f* = 0.76 mmol·g^{-1}). The chain was elongated by sequentially coupling Fmoc-Ile-OH, Fmoc-Leu-OH, Fmoc-Glu(O*t*Bu)-OH, Fmoc-Glu(O*t*Bu)-OH, Fmoc-His(trt)-OH, Fmoc-Asn(trt)-OH, Fmoc-Leu-OH, Fmoc-Asn(trt)-OH, Fmoc-Leu-OH, Fmoc-Asp(O*t*Bu)-OH by using HOBt and DIC as a coupling reagents (fourfold molar

excess) in DMF for 2 h at room temperature. The completion of all couplings was confirmed with a negative ninhydrin test result. After 10 amino acid couplings the resin was spliced three portions, two of them were used for the synthesis of the peptide with 10 amino acid residues and the other portion was used for forwards synthesis. After the last amino acid coupling for each sequence, the Fmoc protective group was removed with 20% piperidine in DMF and the amino terminus was acetylated or modified by the introduction of 5-(6)-carboxyfluorescein. For acetylated peptide, the resin was treated with Ac_2O (4 equiv.; 2 times until the ninhydrin test was negative) for 2 h at room temperature. The treatment of the resin with 5-(6)-carboxyfluorescein (4 equiv.), HOBt (4 equiv.), and DIC (4 equiv.) in DMF for 6 h at room temperature (until the ninhydrin test was negative) afforded the corresponding fluorescein-labeled peptides. Finally, the peptides were cleaved from the resin and the protective groups at amino acid side chains were removed with a cocktail TFA/TIS/H_2O/EDT (94:1:2.5:2.5) for 3 h at room temperature. The filtrated were collected and the solvents were removed under N_2 current. Each product was precipitated by addition of the cleavage solution to cold Et_2O or methyl *t*-butyl ether (4 °C). After centrifugation (4000 min^{-1}, 3 min) the upper phase was removed from the vial, diethyl ether or methyl-*t*-butyl ether was added, the solid was washed for 5 min in an ultrasonic bath, and the vial was centrifuged. The remaining solid was dissolved in CH_3CN/H_2O (1:4, *v*/*v*) and lyophilized. After that, each peptide was purified by reversed phase HPLC and isolated as a white solid after lyophilization in yield 45%.

Fluo-Ie (Fluorescein-DNLNHEELIQ): HPLC purity: 100% (UV, t_R = 18.3 min). ESI-MS (electrospray ionization, positive mode): *m*/*z* calcd for $C_{72}H_{92}N_{15}O_{36}$: 1582.6332 $[M + H]^+$; found: 1582.6327.

1.6. Fluo-If (Fluorescein-NDNLEE) and Fluo-Ig (Fluorescein-DNLEE)

2-Chloro-2-trityl chloride resin (200 mg, f = 1.18 mmol·g^{-1}) placed in a 10 mL polypropylene syringe fitted with a polyethylene filter disk was treated with Fmoc-Glu(O*t*Bu)-OH (410 mg, 0.67 mmol, 4 equiv.) and with diisopropylethylamine (DIPEA; 117 μL, 0.67 mmol, 4 equiv.) in DMF for 15 min at room temperature. Subsequently, an additional portion of DIPEA (235 μL, 1.52 mmol, 8 equiv.) was added and the reaction mixture was stirred for 18 h at room temperature. The resin was filtered off, washed three times with DMF, CH_2Cl_2 and Et_2O, and dried under high vacuum. When the reaction was finished, the loading was calculated by UV spectroscopy (f = 0.87 mmol·g^{-1}). The chain was elongated by sequentially coupling Fmoc-Glu(O*t*Bu)-OH, Fmoc-Leu-OH, Fmoc-Asn(trt)-OH, Fmoc-Asp(O*t*Bu)-OH and for Fluo-pepIf again Fmoc-Asn(trt)-OH, by using HOBt and DIC as coupling reagents (fourfold molar excess) in DMF for 2 h at room temperature. The completion of all couplings was confirmed with a negative ninhydrin test result. After 10 amino acid couplings the resin was spliced three portions, two of them were used for the synthesis of the peptide with 10 amino acid residues and the other portion was used for forwards synthesis. After the last amino acid coupling for each sequence, the Fmoc protective group was removed with 20% piperidine in DMF and the amino terminus was acetylated or modified by the introduction of 5-(6)-carboxyfluorescein. For acetylated peptide, the resin was treated with Ac_2O (4 equiv.; 2 times until the ninhydrin test was negative) for 2 h at room temperature. The treatment of the resin with 5-(6)-carboxyfluorescein (4 equiv.), HOBt (4 equiv.), and DIC (4 equiv.) in DMF for 6 h at room temperature (until the ninhydrin test was negative) afforded the corresponding fluorescein-labeled peptides. Finally, the peptides were cleaved from the resin and the protective groups at amino acid side chains were removed with a cocktail TFA/TIS/H_2O/EDT (94:1:2.5:2.5) for 3 h at room temperature. The filtrated were collected and the solvents were removed under N_2 current. Each product was precipitated by addition of the cleavage solution to cold Et_2O or methyl *t*-butyl ether (4 °C). After centrifugation (4000 min^{-1}, 3 min) the upper phase was removed from the vial, diethyl ether or methyl-*t*-butyl ether was added, the solid was washed for 5 min in an ultrasonic bath, and the vial was centrifuged. The remaining solid was dissolved in CH_3CN/H_2O (1:4, *v*/*v*) and lyophilized. After that, each peptide was purified by reversed phase HPLC and isolated as a white solid after lyophilization in yield between 45% and 55%.

Fluo-If (Fluorescein-NDNLEE): HPLC purity: 100% (UV, t_R = 17.5 min). ESI-MS (electrospray ionization, positive mode): *m*/*z* calcd for $C_{49}H_{55}N_8O_{21}$: 1091.3476 $[M + H]^+$; found: 1091.3465.

Fluo-Ig (Fluorescein-DNLEE): HPLC purity: 100% (UV, t_R = 17.5 min). ESI-MS (electrospray ionization, positive mode): *m*/*z* calcd for $C_{45}H_{48}N_6O_{19}$: 976.2974 $[M + H]^+$; found: 976.2969.

2. Fluorescence Polarization Assay

2.1. Q Determination

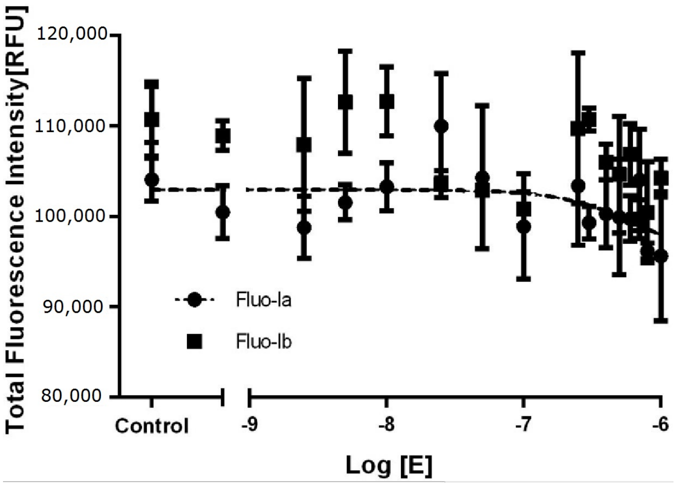

(**a**)

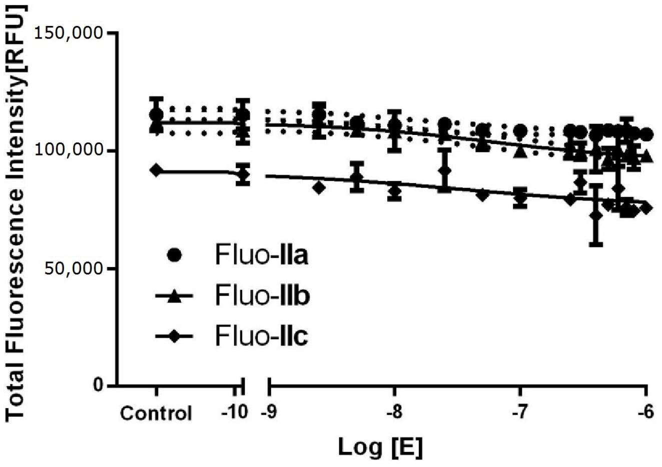

(**b**)

Figure S1. *Q* determination. The peptides **Fluo-Ia,b** and **Fluo-II(a–c)** were used in concentration of 10 nM with increasing concentration of Epsilon (from 0.05 nM to 2.5 μM) in assay buffer (HEPES 10 mM, pH 7.5 with 0.1% Tween-20). The total fluorescence intensity (RFU) was plotted for every pair protein/peptide as a function of the logarithm of the protein concentration. Binding curves were fitted with the aid of GraphPad Prism software 4 for Windows (GraphPad, San Diego, CA, USA) with a sigmoidal dose-response model (4 PL) and obtained from the maximum and minimum value of total fluorescence intensities (FI). The coefficient between FImin/FImax is the *Q* value. Data are the mean of three independent experiments (*n* = 3) ± SD.

2.2 Study of NaCl and DMSO influence on the binding affinity

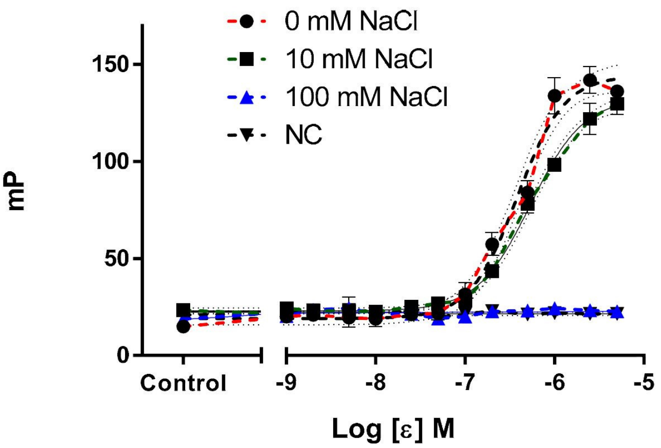

(**a**)

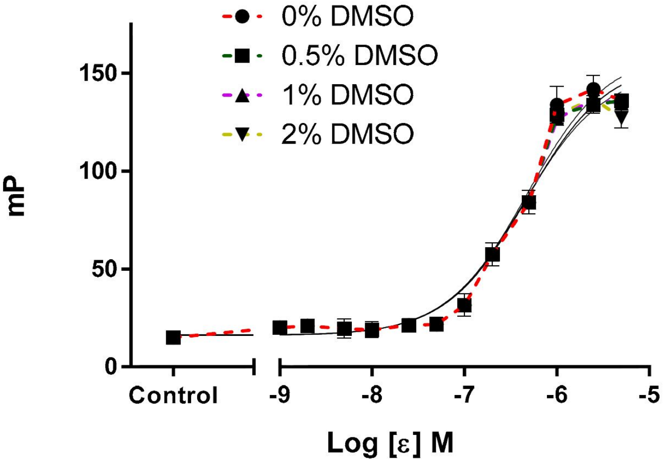

(**b**)

Figure S2. Binding isotherms of fluorescein-labeled peptide **Fluo-Ia** to Epsilon. The peptide **Fluo-Ia** was used in concentration of 10 nM with increasing concentration of Epsilon (from 0.05 nM to 2.5 μM) in assay buffer (HEPES 10 mM, pH 7.5 with 0.1% Tween-20) in presence of increasing concentrations. (**A**) NaCl, 0 (●), 10 (■) or 100 (▲) mM; (**B**) DMSO, 0% (●), 0.5% (■), 1% (▲) and 2% (▼). Data are the mean of three independent experiments (n = 3) ± SD.

2.3. Binding isotherms of peptides Fluo-Id and Fluo-Ie

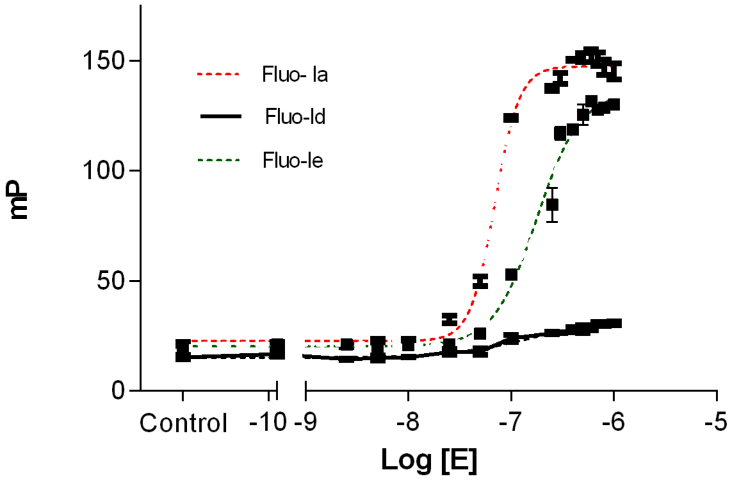

Figure S3. Binding isotherms of fluorescein-labeled peptides **Fluo-Id** and **Fluo-Ie** to Epsilon. In both cases the peptides were used at 10 nM in increasing concentration of Epsilon (0.05 nM to 2.5 μM) in

assay buffer (HEPES 10 mM, pH 7.5 with 0.1% Tween-20). Data are the mean of three independent experiments (*n* = 3) ± SD.

2.4. Z´-factor determination in 384-well assay

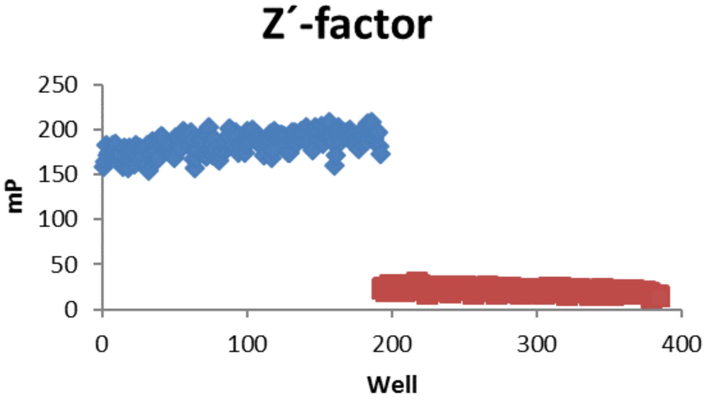

Figure S4. Stability of unbound peptide **Fluo-Ia** and peptide **Fluo-Ia** bound to Epsilon protein in 384-well assay plate. Unbound peptide control (**Fluo-Ia**, 10 nM final concentration) marked in red; bound peptide control (**Fluo-Ia** at 10 nM and Epsilon protein at 500 nM final concentration) marked in blue. Analogous analysis was performed with peptide **Fluo-IIb** (10 nM final concentration) and Epsilon protein (750 nM final concentration).

3. MALDI/TOF analysis of Epsilon

Epsilon protein = antitoxin Epsilon

MGSSHHHHHH SSGLVPRGSH MASMTGGQQM GRGS

MAVTYEKTFE IEIINELSAS VYNRVLNYVL NHELNKNDSQ LLEVNLLNQL
KLAKRVNLFD YSLEELQAVH EYWRSMNRYS KQVLNKEKVA

M+H(avg)= 14,266.15

M+H(avg)= 14,134.96 w/o N-term. Met

(**a**)

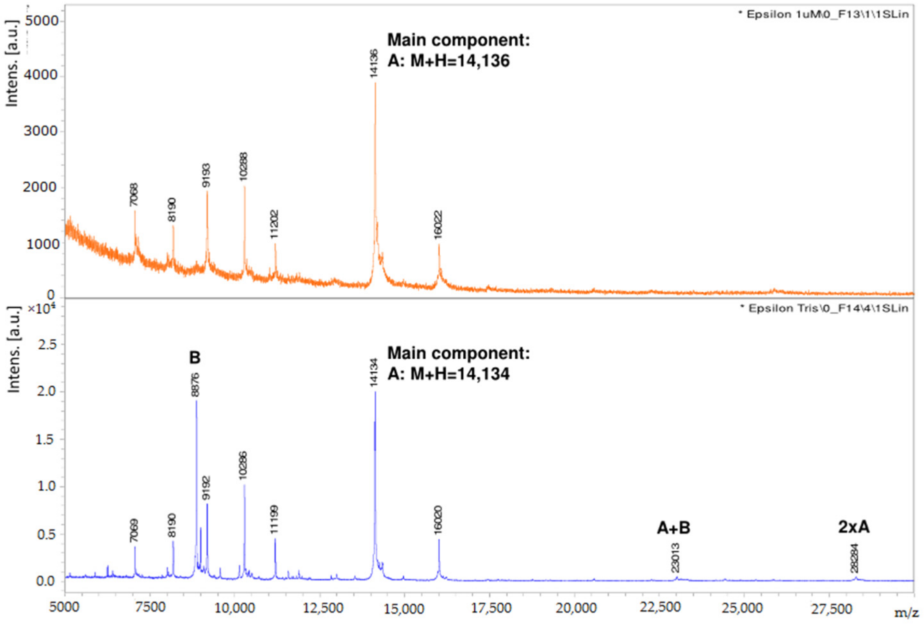

(**b**)

Figure S5. (**a**) Amino acid sequence of the Epsilon protein. (**b**) MALDI/TOF spectra of Epsilon protein at 1 µM obtained using sinapic acid as a matrix.
